# An unusual symptoms caused by huge pseudoaneurysm formation of ascending aorta

**DOI:** 10.1186/1749-8090-10-S1-A337

**Published:** 2015-12-16

**Authors:** A Klvacek, J Konecny, P Santavy, V Lonsky, M Simek, V Hanak

**Affiliations:** 1 Department of Cardiac Surgery, University Hospital Olomouc, I.P. Pavlova 185/6, 779 00 Olomouc, Czech Republic

## Background/Introduction

We are presenting a 33-year-old patient with huge pseudoaneurysm formation of the ascending aorta, developing after type A acute aortic dissection repair.

## Aims/Objectives

This man 10 years ago underwent surgery for acute aortic dissection type Stanford A. During this surgery the ascending aorta including the aortic valve had been replaced. The regular follow-up by echocardiography was performed once a year. After 10 years without any problem, the patient began to suffer from atypical problems - repeated respiratory infections and superior vena cava syndrome. The CTA shown huge pseudoanerysm of the ascending aorta which was oppressing superior vena cava, both main bronchi and esophagus. This finding was the indication for redo surgery. During redo surgery the total suture line dehiscence of distal anastomosis between vascular prosthesis and native aortic arch was found. The blood stream leaking through this dehiscence formed pseudoanerysm sac. The aortic root with mechanical prosthesis was found intact. The supracoronary ascending aorta replacement was performed. Four days after surgery the cardiac tamponade was diagnosed. On operating two small leaks between native aortic arch and vascular graft was directly sutured. Further progress was uneventful.

## Method

Case report.

## Results

Echocardiography and CTA shown a good function of the prosthesis. Fifteen days after surgery the patient was discharged home.

## Discussion/Conclusion

Suture line dehiscence and pseudoaneurysm formation is one of the leading causes of late reoperation after surgical repair of acute type A aortic dissection. Redo surgeries are connected with high risk of death. Therefore timing and good preoperative imagination are very important.

## Consent

Written informed consent was obtained from the patient for publication of this abstract and any accompanying images. A copy of the written consent is available for review by the Editor of this journal.

